# An eFP browser for visualizing strawberry fruit and flower transcriptomes

**DOI:** 10.1038/hortres.2017.29

**Published:** 2017-06-21

**Authors:** Charles Hawkins, Julie Caruana, Jiaming Li, Chris Zawora, Omar Darwish, Jun Wu, Nadim Alkharouf, Zhongchi Liu

**Affiliations:** 1 Department of Cell Biology and Molecular Genetics, University of Maryland, College Park, MD 20742, USA; 2 Centre of Pear Engineering Technology Research, Nanjing Agricultural University, Nanjing 210095, China; 3 Department of Computer and Information Sciences, Towson University, Towson, MD 21252, USA

## Abstract

Wild strawberry *Fragaria vesca* is emerging as an important model system for the cultivated strawberry due to its diploid genome and availability of extensive transcriptome data and a range of molecular genetic tools. Being able to better utilize these tools, especially the transcriptome data, will greatly facilitate research progress in strawberry and other *Rosaceae* fruit crops. The electronic fluorescent pictograph (eFP) software is a useful and popular tool to display transcriptome data visually, and is widely used in other model organisms including *Arabidopsis* and mouse. Here we applied eFP to display wild strawberry RNA sequencing (RNA-seq) data from 42 different tissues and stages, including various flower and fruit developmental stages. In addition, we generated eight additional RNA-seq data sets to represent tissues from ripening-stage receptacle fruit from yellow-colored and red-colored wild strawberry varieties. Differential gene expression analysis between these eight data sets provides additional information for understanding fruit-quality traits. Together, this work greatly facilitates the utility of the extensive transcriptome data for investigating strawberry flower and fruit development as well as fruit-quality traits.

## Introduction

The flower and the fruit represent the most complex organs of flowering plants, made up of diverse tissue types and serving different functions for plant reproduction. Further, the products of flowers, such as seeds and fruits, are major sources of food and feed, contributing to the majority of agricultural output. Therefore, understanding how different floral and fruit tissues develop lays the foundation for agricultural research for enhancing crop yields.

One powerful research tool that is routinely used in plant research is the genome-wide profiling of gene expression. Older technologies such as microarrays and newer technologies such as next-generation sequencing (NGS) are generating an ever-increasing body of transcriptome data sets. Although most of these data are made publicly available, they are underutilized because of the enormous volume of the data and the inaccessibility of the data format due to limited computational knowledge and training of most plant scientists. There is considerable value, therefore, in a simple, web-based interface that allows existing transcriptome data sets to be explored in a visual manner.

The electronic fluorescent pictograph software (eFP) was developed at the University of Toronto in order to facilitate visual exploration of gene expression.^1^ The function of the software is to display a cartoon image depicting various tissue types, each colored in with a hue indicating the level of expression of a queried gene. For instance, diagrams of *Arabidopsis* showing different tissues and organs at various stages of development; each diagram displays a different orange hue reflecting organ-level expression (http://bar.utoronto.ca).^1^ Since then, numerous other species, including poplar, medicago, soybean, potato, tomato, *Eutrema*, maize, rice, barley, triticale, *Physcomitrella*, mouse and human, have had eFP browsers developed (http://bar.utoronto.ca),^2,3^ encompassing a large number of tissues and experiments. This attests to the usefulness of the tool.

Currently, there is no such eFP browser available for strawberry despite the availability of extensive RNA sequencing (RNA-seq) data for flower and early-stage fruit development in the wild strawberry *Fragaria vesca*,^4,5^ which is being developed as a model system for the octoploid garden strawberry *F. ananassa* as well as other *Rosaceae* species.^6^ In addition to diploidy (2*n*=2*x*=14), *F. vesca*’s genome is small (240 Mb haploid genome) and contains 34 809 annotated genes.^6^ To complement previous RNA-seq data on flower and early-stage fruit development,^4,5^ we generated additional sets of RNA-seq data from ripening fruit (receptacle) tissues. As fruit ripening is of significant interest to researchers as well as to the strawberry fruit industry, the generation of this new RNA-seq data set enables comprehensive understanding of gene expression including stages more relevant to fruit quality.

More significantly, we established an *F. vesca* eFP browser that enables visualization of both previous RNA-seq data sets on flower and early-stage fruit development^4,5^ and this new RNA-seq data set on ripening-stage receptacle. With a combined total of 46 different tissues, each with two biological replicates (46×2=92 RNA-seq libraries), this eFP browser for wild strawberry enables intuitive visualization and interpretation of the gene expression data across a wide range of finely dissected flower and fruit tissues at different developmental stages and facilitates comparative studies of gene expression in different fruit crops. The searchable server is hosted at http://mb3.towson.edu/efp/cgi-bin/efpWeb.cgi and serves as an important resource for the fruit crop community.

## Materials and methods

### Green- and white-stage fruit tissue collection, RNA extraction and RNA-seq data generation

*F. vesca* plants were grown in a growth chamber with 16-h light at 25 °C and 8-h dark at 20 °C. Receptacle fruit tissues (with achenes removed) were collected from Ruegen F7-4 and Yellow Wonder 5AF7 (YW), which are both 7th generation inbred lines.^7,8^ Fruits were collected at two different stages: green stage and white (or turning) stage. The stages were determined based on days post anthesis (DPA), color of the receptacle and the distance between achenes on the receptacle. The green stage is at ~15 DPA and the turning stage is at ~22 DPA. At least three fruits were combined to form one biological replicate, and two biological replicates were obtained for each tissue.

Total RNAs were extracted according to the Cetyl trimethyl ammonium bromide (CTAB) method described by Gasic *et al.*^9^ with minor modifications. Briefly, 0.5 g of combined fruits were ground in a mortar with liquid nitrogen, and then transferred into a 2 ml microfuge tube containing 1 ml 2% CTAB buffer (2% CTAB; 2% polyvinylpyrrolidone, PVP K-30; 100 mM Tris-HCl, pH 8.0; 25 mM EDTA; 2 M NaCl; 0.5 g l^−1^ spermidine (free acid)) with 50~70 μl β-mercaptoethanol added prior to use. Samples were incubated for 30 min at 65 °C and vortexed every 5 min. After centrifugation at 13 000 *g* for 10 min at 4 °C, the supernatant was transferred to a new 1.7 ml microfuge tube and then mixed with an equal volume of chloroform/isoamyl alcohol (24:1), immediately vortexed for 5 min, and then centrifuged for 10 min at 12 000 *g* at 4 °C. The chloroform/isoamyl alcohol extraction was repeated once more. Two-third volume of 10 M LiCl was added to each tube and mixed by inversion, and then each tube was stored at −20 °C for 8–12 h. Tubes were centrifuged at 13 000 *g* for 30 min at 4 °C. RNA, in the pellet, was washed twice with 70% ethanol and dried in a clean hood for 2 min, and then the pellet was dissolved in 15 μl diethylpyrocarbonate-treated water. Finally, on column DNase digestion with the RNase-free DNase set (Qiagen, Frederick, MD, USA) was performed to remove genomic DNA. The quantity and quality of RNAs were determined using the Nanodrop 2000c Spectrophotometer (ThermoFisher, Waltham, MA, USA).

### RNA-seq data processing and analysis

RNA-seq data were generated from the Illumina Hiseq2000 (prior study) and HiSeq4000 (this study) instruments, producing 51 bp single-end reads. The flower and early-stage fruit data have previously been published.^4,5^ The raw data were mapped against the *F. vesca* Genome v2.0.a1.^10^ using Bowtie 2 and gene expression levels were normalized to give RPKM (reads per KB per million) values used directly in the eFP display (Supplementary Data S1).

**Figure 4:**
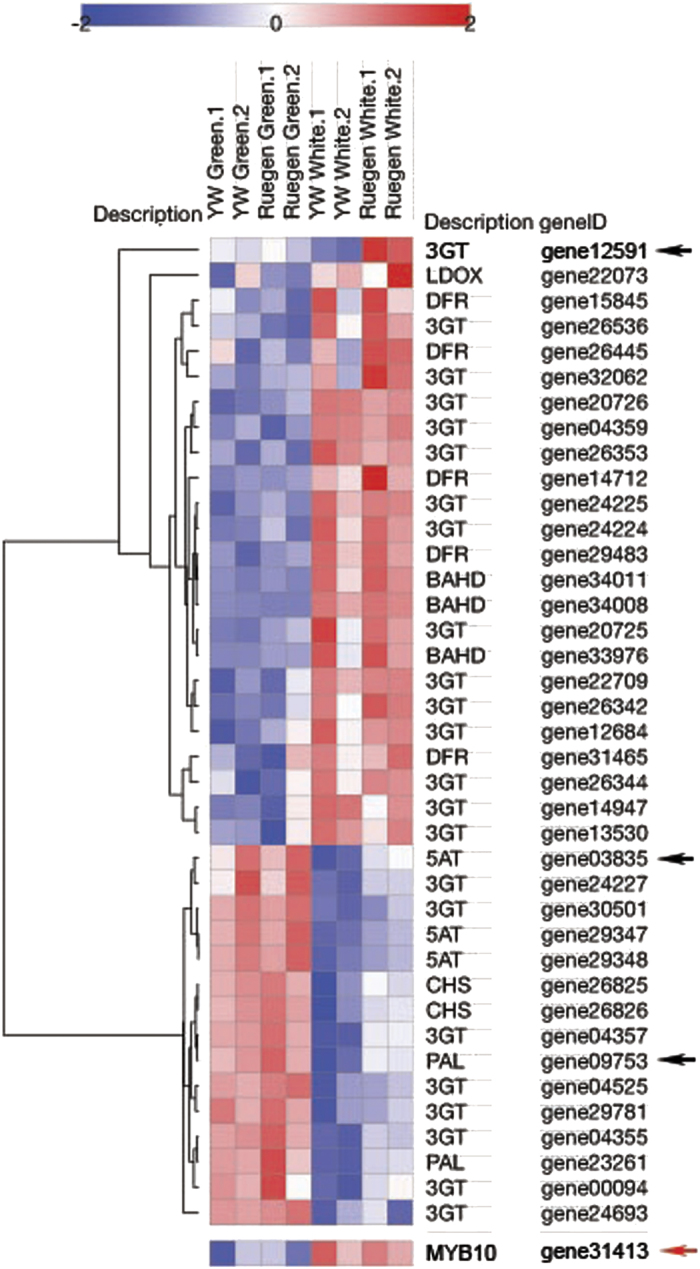
Expression of differentially expressed flavonoid/phenylpropanoid biosynthetic genes. Heatmap displaying *z*-score of log_2_ RPKM for flavonoid/phenylpropanoid genes with RPKM >5 in at least one tissue. All shown genes are differentially expressed between green and white sample sets at false discovery rate (FDR)-corrected *P*⩽0.01. Expression of *FveMYB10* (red arrow) is also shown. Three genes that are differentially expressed between YW5AF7 and Ruegen at the white stage are indicated with black arrows. Gene descriptions: *3GT*, anthocyanidin 3-O-glucosyltransferase; * BAHD*, BAHD acyltransferase; *CHS*, chalcone synthase; *DFR*, dihydroflavonol-4-reductase;*LDOX*, leucoanthocyanin dioxygenase; *PAL*, phenylalanine ammonia lyase; *5AT*, anthocyanin 5-aromatic acyltransferase.

Differential expression analysis was carried out using the DESeq2 package in R.^11^ Genes for which the Benjamini–Hochberg-adjusted *P* values were ⩽0.01 and the log_2_ fold change was ⩾1 or ⩽−1 were considered significantly differentially expressed (DE) between samples. The heatmap showing expression of genes in the anthocyanin biosynthetic pathway in Figure 4 was generated using the Morpheus webtool available from the Broad Institute (https://software.broadinstitute.org/morpheus/).

### Drawing of tissue types

The fruit and flower diagrams in eFP were drawn based on similar photographic figures from Kang *et al.*^4^ and Hollender *et al.*^12^ using the Inkscape vector illustration software and the GIMP image-editing software. Then, the diagrams were exported in a TGA format as required by eFP. The eFP software used is version 1.6.0, downloaded from https://sourceforge.net/projects/efpbrowser/, where it is available under the GNU General Public License v2.0. Some small modifications were made to the software to support our RNA-seq data.

### Scripts

The new script, import_transcriptome.py, functions to import transcriptome data from Excel spreadsheets to a MySQL database for use with eFP. The Excel spreadsheet must be formatted as a grid of expression values, with each column corresponding to one sample and each row corresponding to one gene. The first row should have the sample names and the first column should have the gene identifiers (these are what will be searched for by the user). The script will read data from only one sheet within the document (the sheet may be specified by name on the command line), but entering data from multiple sheets is possible simply by running the script multiple times, specifying a different sheet each time. The script is written in Python and accepts command-line options (Supplementary Table S3).

The script will ask the user to securely type in the MySQL password for the user specified on the command line. The script adds to any existing data in the database; if data need to be removed first, then this must still be done manually.

### Data submission

The Illumina raw sequence data of all eight samples (Supplementary Table S1) for the green- and white-stage receptacle fruit have been submitted to the Sequence Read Archive at NCBI (http://www.ncbi.nlm.nih.gov/sra). The accession numbers are SRR5155708 to SRR515515.

## Results

### Description of fruit samples and fruit RNA-seq data sets

**Figure 1:**
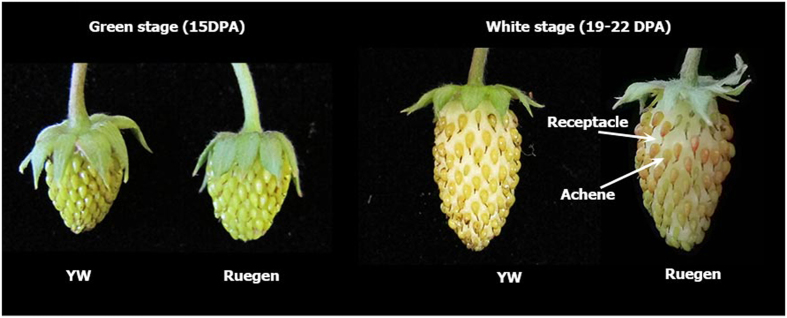
Photos of fruit harvested at green stage (15 DPA) and white stage (19–22 DPA). Both yellow fruit variety (YW5AF7) and red fruit variety (Ruegen F7-4) are shown.

**Figure 2:**
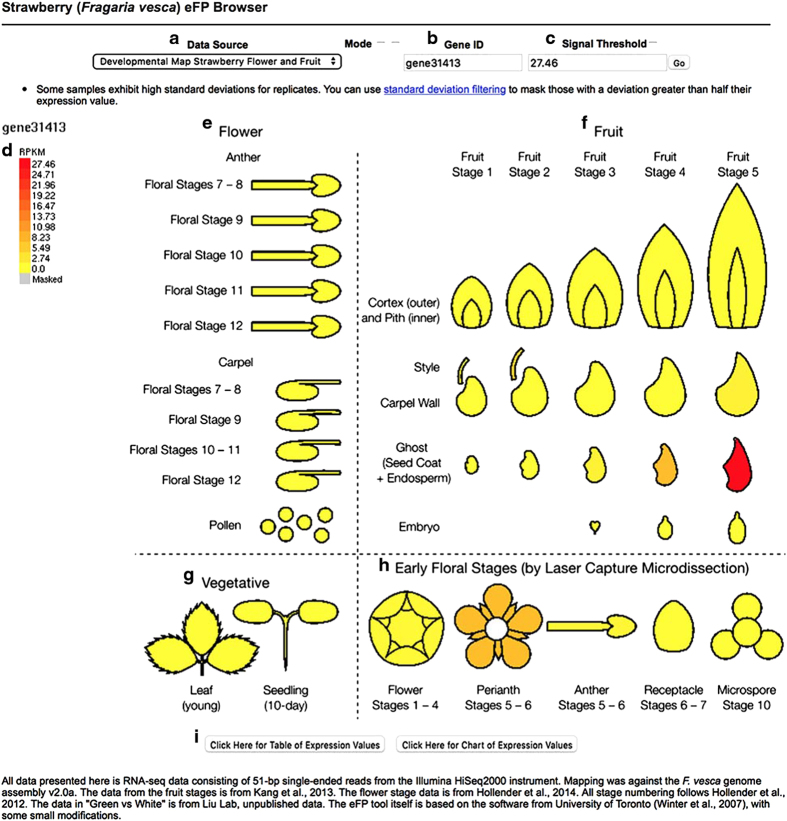
An explanation of the eFP user interface and example output for *FveMYB10* (*gene31413*) during flower and early fruit development. Screenshot of Strawberry eFP showing the user interface elements*. Gene31413* (*FvMyb10*) is shown as an example. Fields along the top allow for different developmental maps to be selected (**a**) and for a gene ID to be queried (**b**). The maximum signal value, used to scale the colors of the image, can be manually adjusted if desired (**c**). The scale bar (in RPKM) is displayed on the left (**d**). The image displayed is divided into sections showing the floral stages (**e**), early-stage fruit development (stages 1–5; **f**), vegetative tissues (**g**) and early floral stages (dissected via laser capture microdissection (LCM); **h**). Buttons along the bottom allow for the data to be presented as a table or bar chart (**i**).

Our prior fruit transcriptome focuses on early-stage fruit development, from stage 1 to stage 5.^4^ Stage 1 is the pre-fertilization stage and stages 2–5 are post-fertilization stages.^4,12^ The fleshy fruit of the strawberry is an accessory fruit in which the stem tip, called the receptacle, develops into the edible fruit (Figure 1). In contrast, the numerous botanical fruits of strawberry, called achenes, dot the surface of the receptacle (Figure 1). Each achene is actually a fertilized ovary consisting of a single seed inside a protective ovary wall. The RNA-seq data are from dissected fruit tissues: each achene is dissected into three subtissues: the protective carpel wall (or ovary wall), the embryo and the ghost (the seed coat and endosperm), whereas the receptacle is dissected into two tissues, cortex and pith (Figure 2f). Supplementary Table 2 lists in detail the specific fruit samples displayed by eFP.

For studying later-stage fruit development such as fruit ripening, a second fruit RNA-seq data set was made in this study. Receptacle tissues were harvested at the green stage and the white (also called turning) stage from both Yellow Wonder (YW5AF7) and Ruegen accessions. The green stage is ~15 DPA (similar to stage 5 described above), whereas the white stage is ~19–22 DPA. White stage is a critical fruit developmental stage when the receptacle fleshy fruit changes color from green to white and the achenes starts turning pink (Figure 1). The two accessions were chosen as Ruegen makes a red fleshy receptacle, whereas the YW5AF7 develops yellow fleshy receptacles. Two biological replicates of each sample were harvested (see Materials and methods and Supplementary Table S1), leading to a total of eight RNA-seq libraries (two accessions each at two fruit developmental stages, and each stage has two biological replicates).

### Description of flower samples and flower RNA-seq data sets

Hollender *et al.*^12^ divided the entire process of flower development into 12 stages from floral primordium initiation to the completion of floral organ development. Older floral stages (stages 7–12) were hand-dissected (Figure 2e) and consist primarily of reproductive tissues (anthers, pollen and carpels). Laser capture microdissection (LCM) was employed to dissect young (stages 1–6) floral tissues (Figure 2h), which are too small for hand dissection. As differences exist in sample isolation, processing and RNA amplification between LCM samples and hand-dissected samples, it difficult to directly compare gene expression levels between LCM and hand-dissected samples.^5^ Therefore, the RNA-seq data from hand-dissected floral tissues were not grouped together with the LCM young floral samples (Figure 2). Supplementary Table S2 lists in detail the specific floral samples displayed by eFP.

### Adapting eFP for diploid strawberry transcriptome

Although the eFP software was previously and primarily designed to display microarray data, it is readily adaptable for RNA-seq data. First, the RNA-seq data were mapped to the *F. vesca* whole-genome assembly version 2.0.a1.^6,10^ The mapped reads in RPKM (reads per KB per million) are in the form of Microsoft Excel files with a column for each sample type and a row for each gene (Supplementary Data S1). In total, 33 673 genes are represented based on *F. vesca* Genome v2.0.a1.^10^ Second, images of different flower and fruit stages (Figure 2) were drawn based on photographs shown in Hollender *et al.*^12^ and Kang *et al.*^4^ Third, a Python script, import_transcriptome.py (Supplementary Table S3), was written to automatically read an Excel file formatted in this way and make the appropriate entries in the MySQL database that eFP uses as its back-end.

The eFP software is written in Python and will run on an Apache web server that supports Common Gateway Interface (CGI). It works by taking a pre-drawn template figure depicting the tissues with each tissue filled in with some unique color, and replacing each unique color with a color that depicts the expression level of the queried gene. The installation is backed by a MySQL database with entries containing the measured RPKM for each combination of gene ID and tissue. When the user queries a gene, MySQL commands are generated to query the database for each of the tissues depicted in the figure to be displayed, and for each tissue a color is generated based on a linear scale from zero (yellow) to the maximum expression of that gene among the tissues depicted (red). The software then replaces each unique color in the template with the color corresponding to the expression level of the gene. An XML-based configuration file for each figure specifies what tissues are in the figure and which unique color in the template figure corresponds to which tissue.

The MySQL database must be set up and populated manually beforehand, as eFP does not possess the capability to create and configure this database itself, or to input any data into it. The new import_transcriptome.py script can automate much of the data entry step if the transcriptome data are available in the form of a compatible Excel document. This set-up is flexible enough that new samples from new tissue types may be added to the database by re-running the script on another Excel file containing the new data. The template image used by the software to generate the diagrams and the developmental map descriptor file must also be updated to show the new samples.

### Interpretation of strawberry eFP images

The eFP is hosted at the Strawberry Genome Resource server http://bioinformatics.towson.edu/strawberry/.^13^ It can be directly accessed from http://mb3.towson.edu/efp/cgi-bin/efpWeb.cgi. The website is accessible by anyone. As illustrated in Figure 2, the line drawing of each tissue is colored a shade from yellow to red based on the specific RPKM value of the queried gene. An option is available to gray out tissues for which the expression level of the queried gene is less than its s.d. (based on the two replicates), a condition that may indicate that the expression measurement is unreliable.

**Figure 3:**
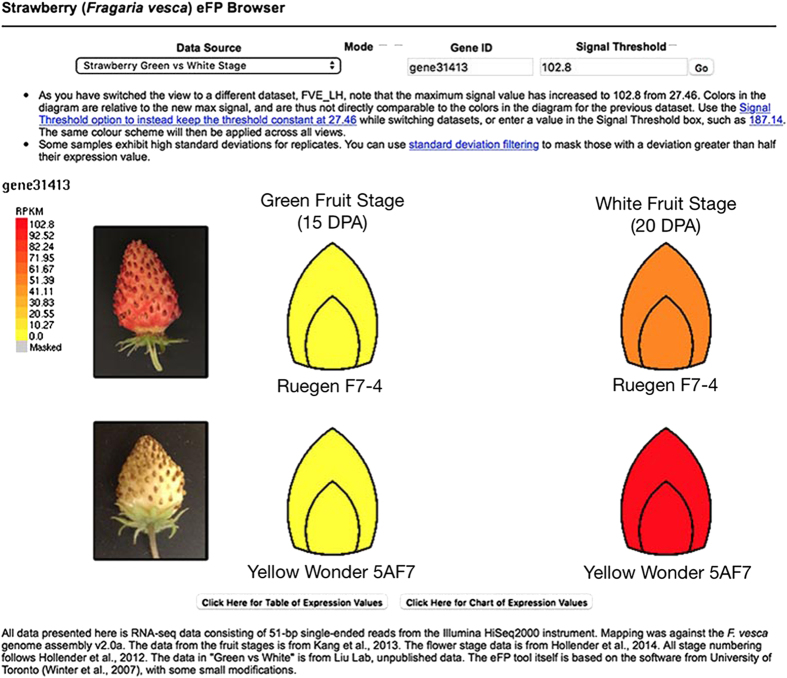
Example output for *FveMYB10 (gene31413*) during fruit-ripening stage. Receptacle fruit tissues from two different varieties of *F. vesca* are shown. The Ruegen F7-4 makes red fruit, whereas the Yellow Wonder 5AF7 makes yellow fruit because of a missense mutation in *FveMYB10 (gene31413)*. RPKM values for *FveMYB10* (*gene31413)* are higher at the white stage in YW as compared to Ruegen, but this difference is not statistically significant.

There are two different eFP pages: one page describes all flower RNA-seq data as well as the early-stage fruit RNA-seq data (Figure 2). The second page is focused on the ‘white’ stage fruit development in the red (Ruegen) and yellow (YW5AF7) fruit accessions (Figure 3).

By default, the color scale in the diagram is scaled from zero to the maximum observed expression for the queried gene (see the left scale bar in Figure 2d). However, a user may choose to enter a different maximum value manually in the field marked ‘Signal Threshold’ (the checkbox above it enables this feature); in this case, the software uses the maximum entered by the user. By clicking the buttons ‘Click Here for a Table of Expression Values’ at the bottom of the page, a table listing exact expression values of the queried gene in each of the samples will be generated. Alternatively, one can click ‘Click Here for a Chart of Expression Values’, a histogram depicting that the same data will be generated.

A gene’s ID (geneXXXXX) must be used in query the Strawberry eFP Browser. This gene ID is a standard identifier for *F. vesca* genes. For example, the transcription factor *FveMYB10* has the gene ID ‘gene31413’. An easy way to retrieve the gene ID is to search the Plaza database (http://bioinformatics.psb.ugent.be/plaza/versions/plaza_v3_dicots/) using a gene’s descriptive name. If a gene cannot be found by its name, its DNA sequence can be used to BLAST within the Plaza to identify the corresponding gene. Once identified, the Plaza information page includes information about the gene including its unique gene ID.

### *FveMYB10 (gene31413)* expression provides insights into fruit pigment development

We previously reported that a single nucleotide polymorphism causing a W to S substitution in the R2 DNA-binding domain of *FveMYB10 (gene31413)* was responsible for the fruit color difference in most yellow versus red fruit *F. vesca* accessions.^8^ The yellow fruit accession YW5AF7 contains S residue, whereas the red fruit Ruegen contains W residue in the R2 DNA-binding domain of FveMYB10. We queried this gene using eFP (Figures 2 and 3). First, expression of *FveMYB10* appears very low during flower development and early-stage fruit development (Figure 2). However, there is a surge of *FveMYB10* expression at stage 5 (~15 DPA) ghost. Each ghost consists of a seed coat and endosperm tissue, and is encased inside an ovary wall, together forming the achene. The function of this surge of *FveMYB10* expression at stage 5 ghost is not known.

To understand strawberry fleshy fruit ripening, later-stage fruit development is shown in the second eFP page (select from the ‘Data Source’ drop down menu on the top of the eFP webpage), which displays expression in both green- and white-stage receptacle fruit (Figure 3). *FveMYB10* is highly expressed at the white stage in both yellow (YW5AF7) and the red (Ruegen) strawberries, indicating that *FveMYB10* expression coincides with the time when the receptacle fruit starts changing color. Further, the *FveMYB10* transcript level is not reduced in the yellow fruit despite harboring a mutant allele (Figure 3). This is not surprising, given that the mutant allele in YW5AF7 is a missense mutation that should not affect its RNA expression. Although *FveMYB10* appeared to be expressed at a higher level in the YW5AF7 fruit than Ruegen fruit, this expression level difference was not statistically significant based on DESeq2 analysis.

### Differential gene expression analysis identified downstream targets of *FveMYB10*

We generated the green- and white-stage fruit receptacle RNA-seq data set (Figure 1 and Supplementary Table S1) for display at eFP as well as for identification of genes whose specific upregulation at the white stage suggests critical function during strawberry ripening. We performed differential gene expression analysis using the DESeq2 package in R^11^ between the green stage and white stage RNA-seq data, using combined data sets for YW and Ruegen; this analysis aims to identify developmentally regulated genes. Several thousand DE genes between green and white stages were identified (Supplementary Data S2); these DE genes are likely involved in various aspects of ripening process including production of phenolic metabolites, anthocyanin biosynthesis and cell wall softening. Figure 4 illustrates expression of a subset of these DE genes with functions in the flavonoid/phenylpropanoid biosynthesis pathway. Interestingly, genes in the upper cluster are induced at the white stage, whereas those in the lower cluster are expressed at the green stage but downregulated at the white stage (Figure 4). *FveMYB10* expression closely aligns with the top cluster as it may be involved in activating the top cluster of genes (Figure 4).

Next, we compared receptacle RNA-seq data between Ruegen and YW5AF7 at the white stage only to identify downstream targets of *FveMYB10,* as YW5AF7 is defective in *FveMYB10*. This comparison resulted in 518 DE genes (Supplementary Data S3), three of which are predicted to function in the anthocyanin biosynthetic pathway and are among the developmentally regulated genes shown in the heatmap (Figure 4). These genes are anthocyanidin 3-O-glucosyltransferase (*3GT;* gene12591), phenylalanine ammonia lyase (*PAL; gene09753*) and anthocyanin 5-aromatic acyltransferase (*5AT; gene03835*). *gene12591* is of particular interest as it is the most highly DE gene overall, with 70-fold greater expression in Ruegen versus YW, indicating that it may be a bona fide downstream target of *FveMYB10*. As a putative 3GT (sometimes referred to as UDP-glucose:flavonoid-3-*O*-glucosyltransferase; *UFGT*), gene12591 catalyzes the final step in the synthesis of pelargonidin 3-O-glucoside, the main anthocyanin responsible for the red pigment in strawberry.

### The expression pattern of an auxin biosynthesis gene, *FveYUC10 (Gene27796)*, provides insight into fruit set

The ability to analyze gene expression profiles during fruit development will help elucidate the developmental processes that lead to the formation of the strawberry’s unusual fruit and identify candidate genes for functional studies. Fertilization-induced auxin biosynthesis from the achene is known to be critical for fruit set. Removing the achenes from the receptacle blocks fruit set, whereas exogenous application of the plant hormone auxin can restore fruit set even in the absence of achenes.^14^ The achene, however, is a complex organ, consisting of the ovary wall, seed coat, endosperm and embryo, and the specific tissue responsible for auxin biosynthesis needed for fruit set is not yet determined. Our prior study found higher levels of expression in the ghost for auxin and gibberellic acid (GA) biosynthesis genes.^4^

**Figure 5:**
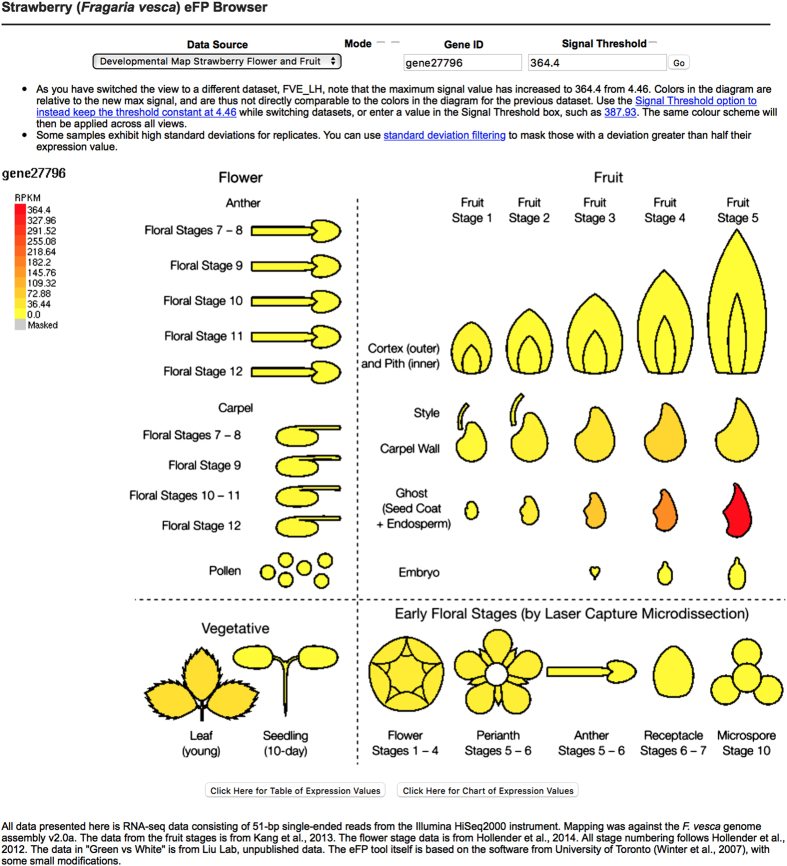
Expression of *FveYUC10 (gene27796)* during flower and early-fruit development. *FveYUC10*, encoding an flavin monooxygenase for tryptophan-dependent auxin biosynthesis, is expressed in the ghost (endosperm+seed coat) post fertilization. This supports the identification of this tissue as the source of auxin in fruit set.^4^

Using eFP, we are able to visualize tissue-specific expression of an auxin biosynthesis gene, *FveYUC10* (*Gene27796; *Figure 5), which encodes a member of the *YUCCA (YAC)* family of flavin monooxygenases that function in tryptophan-dependent auxin biosynthesis. As shown (Figure 5), *FveYUC10* is preferentially expressed in the ghost (seed coat+endosperm) post fertilization (stages 3–5), supporting that auxin biosynthesis post fertilization mainly occurs in the endosperm and seed coat for fruit set.^4^ The surge of auxin made in endosperm/seed coat may then be transported into the receptacle to initiate fleshy fruit development.

## Discussion

### eFP is an important tool for strawberry flower and fruit

In the post-genomic era, tools for quickly and easily examining high-throughput data sets in an intuitive and visual manner without the need for complex bioinformatics software and the associated skillsets are highly valuable. They are essential to make large, publicly available data sets accessible to many more researchers and to promote cross-disciplinary research and collaboration between data scientists, biologists and horticulturists. Even where the skillsets to use technical software are available, repeated analyses of existing data are time-consuming and represent an unnecessary duplication of effort. A simple and convenient interface for data exploration helps to alleviate these problems. The fact that the same software, eFP, already hosts data sets from species such as *Arabidopsis* and *Medicago* will also facilitate comparisons between strawberry and species with more ‘conventional’ fruit architectures.

The wild strawberry eFP Browser described here serves to allow easy exploration of the extensive *F. vesca* flower and fruit transcriptome data sets both from previous studies and the current study. This visualization tool may help encourage the broader community to use and explore these RNA-seq data sets, facilitate investigations into the genes that control flower and fruit development, and stimulate hypothesis formulation. It makes it easier to compare gene expression among different species and can be easily expanded to host flower and fruit RNA-seq data from other species. Having this tool will also help advance *F. vesca* as a model species. Since *F. vesca* is the model member of *Rosaceae*, this work lays the foundation for the possible extension of this eFP to include other *Rosaceae* species such as apple and peach, for which abundant RNA-seq data are also available, for example, refs 15,16,17

### Regulation of *FveMYB10* and its downstream genes for fruit-quality traits

Earlier reports in *F. vesca* and *F. ananassa* have already demonstrated the critical role of *MYB10* in red pigment formation in strawberry.^18–20^ Transgenic plants overexpressing *MYB10* or showing silenced *MYB10* displayed corresponding up- or downregulation of genes in the known flavonoid/phenylpropanoid pathway.^18–20^ A missense mutation in the *FveMYB10* causes the yellow-colored fruit in many of the wild strawberry varieties.^8^ The eFP diagram nicely displays the upregulation of *FveMYB10* expression at the white stage in the receptacle, which is shortly before red pigmentation begins to appear (Figure 1). This timing is consistent with the key role *MYB10* has in the activation of the anthocyanin pathway.

However, our results appear to differ from prior studies in comparing gene expression changes between *MYB10* RNA interference (RNAi)- silenced versus nonsilenced *F. ananassa* fruit and between red and yellow-fruited *F. vesca*.^18,19,21^ In the prior studies, expression of multiple early- and late-regulated biosynthesis genes in the flavonoid/phenylpropanoid pathway was reduced in transgenic *MYB10* RNAi or yellow-fruited strawberry. In contrast, our RNA-seq data showed a limited number of biosynthesis genes whose expression was reduced in the yellow-fruited strawberry. Strikingly, *gene12591*, a 3GT, which catalyzes the final step of the anthocyanin pathway for the synthesis of pelargonidin 3-O-glucoside), showed significant change with a 70-fold increase in the red-fruited Ruegen when compared with yellow-fruited YW, suggesting that *gene12591* is a direct target of *FveMYB10* and mediates the effect of *FveMYB10* on fruit color. This is consistent with previous report about this gene being a direct target of *FveMYB10*. Specifically, a luciferase transactivation assay in tobacco showed that *FveMYB10* can activate the promoter of *gene12591*.^18^ The difference between our data and previous studies that identified larger number of DE anthocyanin biosynthesis genes is likely due to difference in the fruit samples used in the study. The previous studies used red-stage fruit or pooled fruit stages (from turning to red), which represent gene expression differences at later stages of fruit ripening. In contrast, our study uses the white-stage receptacle fruit, which represents the earliest stage of fruit ripening. Hence, our data may reflect the earliest deviation between red and yellow fruit development.

*FveMYB10* may regulate other ripening processes in addition to pigment. By examining other DE genes derived from our study as well as prior studies, one may identify potential target genes of *FveMYB10* that mediate other aspects of fruit-quality traits including flavor and firmness. Interestingly, *FveMYB10* was observed to be upregulated in a second tissue during early-stage fruit development, the stage 5 ghost (Figure 2). Ghost consists of seed coat and endosperm tissues. It may suggest a role of *FveMYB10* in seed coat or endosperm development. The finely dissected fruit and flower tissue types at different stages of development allows researchers to discover previously unknown expression patterns at specific temporal and spatial domains. The eFP web browser reported here makes this important RNA-seq data resource more accessible to the research community.
