# Supplementary material for: An eFP browser for visualizing strawberry fruit and flower transcriptomes
Source: Hortic Res. 2017 Jun 21;4:17029–. doi: 10.1038/hortres.2017.29 (PMC5478792; doi:10.1038/hortres.2017.29)
Supplement: Supplementary Table S2 [file hortres201729-s2.docx]

| **Sample** | **Sample description** | |
| --- | --- | --- |
| **Below are hand dissected flower and early stage fruit tissues** | | |
| Cortex1-1 | Cortex from just open flower, replicate 1 | |
| Cortex1-2 | Cortex from just open flower, replicate 2 | |
| Cortex2-1 | Cortex from the flowers which have been pollinated for about 3 days, replicate 1 | |
| Cortex2-2 | Cortex from the flowers which have been pollinated for about 3 days, replicate 2 | |
| Cortex3-1 | Cortex at about 6 DPA, same age as embryo-3, replicate 1 | |
| Cortex3-2 | Cortex at about 6 DPA, same age as embryo-3, replicate 2 | |
| Cortex4-1 | Cortex at about 9 DPA, same age as embryo-4, replicate 1 | |
| Cortex4-2 | Cortex at about 9 DPA, same age as embryo-4, replicate 2 | |
| Cortex5-1 | Cortex at about 12 DPA, same age as embryo-5, replicate 1 | |
| Cortex5-2 | Cortex at about 12 DPA, same age as embryo-5, replicate 2 | |
| Embryo3-1 | Heart stage embryos,at about 6 DPA, replicate 1 | |
| Embryo3-2 | Heart stage embryos,at about 6 DPA, replicate 2 | |
| Embryo4-1 | Immature cotyledon stage embryos,at about 9 DPA, replicate 1 | |
| Embryo4-2 | Immature cotyledon stage embryos,at about 9 DPA, replicate 2 | |
| Embryo5-1 | Mature embryos which fill up entire ovules,at about 12 DPA, replicate 1 | |
| Embryo5-2 | Mature embryos which fill up entire ovules,at about 12 DPA, replicate 2 | |
| Ghost3-1 | Seeds without embryos inside at about 6 DPA, same age as embryo-3, replicate 1 | |
| Ghost3-2 | Seeds without embryos inside at about 6 DPA, same age as embryo-3, replicate 2 | |
| Ghost4-1 | Seeds without embryos inside at about 9 DPA, same age as embryo-4, replicate 1 | |
| Ghost4-2 | Seeds without embryos inside at about 9 DPA, same age as embryo-4, replicate 2 | |
| Ghost5-1 | Seeds without embryos inside at about 12 DPA, same age as embryo-5, replicate 1 | |
| Ghost5-2 | Seeds without embryos inside at about 12 DPA, same age as embryo-5, replicate 2 | |
| Ovule1-1 | Ovules from just open flower, replicate 1 | |
| Ovule-1-2 | Ovules from just open flower, replicate 2 | |
| Ovule2-1 | Seeds from the flowers which have been pollinated for about 3 days, replicate 1 | |
| Ovule2-2 | Seeds from the flowers which have been pollinated for about 3 days, replicate 2 | |
| Pith1-1 | Pith from just open flower, replicate 1 | |
| Pith1-2 | Pith from just open flower, replicate 2 | |
| Pith2-1 | Pith from the flowers which have been pollinated for about 3 days, replicate 1 | |
| Pith2-2 | Pith from the flowers which have been pollinated for about 3 days, replicate 2 | |
| Pith3-1 | Pith at about 6 DPA, same age as embryo-3, replicate 1 | |
| Pith3-2 | Pith at about 6 DPA, same age as embryo-3, replicate 2 | |
| Pith4-1 | Pith at about 9 DPA, same age as embryo-4, replicate 1 | |
| Pith4-2 | Pith at about 9 DPA, same age as embryo-4, replicate 2 | |
| Pith5-1 | Pith at about 12 DPA, same age as embryo-5, replicate 1 | |
| Pith5-2 | Pith at about 12 DPA, same age as embryo-5, replicate 2 | |
| Style-1-1 | Styles and stigmas from just open flowers, replicate 1 | |
| Style-1-2 | Styles and stigmas from just open flowers, replicate 2 | |
| Style2-1 | Style from the flowers which have been pollinated for about 3 days, replicate 1 | |
| Style2-2 | Style from the flowers which have been pollinated for about 3 days, replicate 2 | |
| Wall1-1 | Carpel walls or ovary walls from just open flower, replicate 1 | |
| Wall1-2 | Carpel walls or ovary walls from just open flower, replicate 2 | |
| Wall2-1 | Carpel walls or ovary walls from the flowers which have been pollinated for about 3 days, replicate 1 | |
| Wall2-2 | Carpel walls or ovary walls from the flowers which have been pollinated for about 3 days, replicate 2 | |
| Wall3-1 | Carpel walls or ovary walls at about 6 DPA, same age as embryo-3, replicate 1 | |
| Wall3-2 | Carpel walls or ovary walls at about 6 DPA, same age as embryo-3, replicate 2 | |
| Wall4-1 | Carpel walls or ovary walls at about 9 DPA, same age as embryo-4, replicate 1 | |
| Wall4-2 | Carpel walls or ovary walls at about 9 DPA, same age as embryo-4, replicate 2 | |
| Wall5-1 | Carpel walls or ovary walls at about 12 DPA, same age as embryo-5, replicate 1 | |
| Wall5-2 | Carpel walls or ovary walls at about 12 DPA, same age as embryo-5, replicate 2 | |
| Anther_7-8_A | Anthers from stage 7 or 8 flowers, replicate 1, Arcturis kit | |
| Anther_7-8_B | Anthers from stage 7 or 8 flowers, replicate 2, Arcturis kit | |
| Anther_9_A | Anthers from stage 9 flowers, replicate 1 | |
| Anther_9_B | Anthers from stage 9 flowers, replicate 2 | |
| Anther_10_A | Anthers from stage 10 flowers, replicate 1 | |
| Anther_10_B | Anthers from stage 10 flowers, replicate 2 | |
| Anther_11_A | Anthers from stage 11 flowers, replicate 1 | |
| Anther_11_B | Anthers from stage 11 flowers, replicate 2 | |
| Anther_12_A | Anthers from stage 12 flowers, replicate 1 | |
| Anther_12_B | Anthers from stage 12 flowers, replicate 2 | |
| Carpel_7-8_A | Carpels from stage 7 or 8 flowers, replicate 1, Arcturis kit | |
| Carpel_7-8_B | Carpels from stage 7 or 8 flowers, replicate 2, Arcturis kit | |
| Carpel_9_A | Carpels from stage 9 flowers, replicate 1, Arcturis kit | |
| Carpel_9_B | Carpels from stage 9 flowers, replicate 2, Arcturis kit | |
| Carpel_10_A | Carpels from stage 10 flowers, replicate 1 | |
| Carpel_10_B | Carpels from stage 10 flowers, replicate 2 | |
| Carpel_12_A | Carpels from stage 12 flowers, replicate 1, Arcturis kit | |
| Carpel_12_B | Carpels from stage 12 flowers, replicate 2, Arcturis kit | |
| Pollen_A | Pollen collected from open flowers, replicate 1 | |
| Pollen_B | Pollen collected from open flowers, replicate 2 | |
|  | | |
| **Below are LCM (Laser Capture Microdissection) flower samples** | | |
| Flower_1-4_A | | Pooled tissues of entire flowers from stage 1 to stage 4, replicate 1 |
| Flower_1-4_B | | Pooled tissues of entire flowers from stage 1 to stage 4, replicate 2 |
| Perianth_5-6_A | | Perianths (sepals and petals) from stage 5 or 6 flowers, replicate 1 |
| Perianth_5-6_B | | Perianths (sepals and petals) from stage 5 or 6 flowers, replicate 2 |
| Anther_6-7_A | | Anthers from stage 6 or 7 flowers, replicate 1 |
| Anther_6-7_B | | Anthers from stage 6 or 7 flowers, replicate 2 |
| Receptacle_6-7A | | Developing receptacle from stage 6 or 7 flowers, replicate 1 |
| Receptacle_6-7A | | Developing receptacle from stage 6 or 7 flowers, replicate 2 |
| Microspore_10_A | | Microspores from stage 10 flowers, replicate 1 |
| Microspore_10_B | | Microspores from stage 10 flowers, replicate 2 |
| **Below are vegetative samples** | | |
| Leaf-1 | | Young trifoliate leaves, replicate 1 |
| Leaf-2 | | Young trifoliate leaves, replicate 2 |
| Seedling-1 | | Whole seedlings at 10 days post germination growing in MS medium, replicate 1 |
| Seedling-2 | | Whole seedlings at 10 days post germination growing in MS medium, replicate 2 |
| **Late stage receptacle samples (hand dissected)** | | |
| RG.15d.1 | | Ruegen receptacle tissue at about 15 DPA, replicate 1 |
| RG.15d.2 | | Ruegen receptacle tissue at about 15 DPA, replicate 2 |
| RG.turning.1 | | Ruegen receptacle at ~ 19-21 DPA (white/turning stage), replicate 1 |
| RG.turning.2 | | Ruegen receptacle at ~ 19-21 DPA (white/turning stage), replicate 2 |
| YW.15d.1 | | Yellow Wonder receptacle tissue at ~ 15 DPA, replicate 1 |
| YW.15d.2 | | Yellow Wonder receptacle tissue at ~ 15 DPA, replicate 2 |
| YW.turning.1 | | Yellow Wonder receptacle at ~19-21 DPA (white/turning stage), replicate 1 |
| YW.turning.2 | | Yellow Wonder receptacle at ~19-21 DPA (white/turning stage), replicate 2 |
