# Supplementary material for: An eFP browser for visualizing strawberry fruit and flower transcriptomes
Source: Hortic Res. 2017 Jun 21;4:17029–. doi: 10.1038/hortres.2017.29 (PMC5478792; doi:10.1038/hortres.2017.29)
Supplement: Supplementary Table S3 [file hortres201729-s3.docx]

| **Option** | **Description** |
| --- | --- |
| -h | Show help and usage information |
| -x file.xlsx | Excel workbook (in xlsx format) containing RPKM values. Columns are samples, rows are genes. |
| -s sheet | Name of worksheet to use. Default is "RPKM". |
| -k socket | Socket to connect to the MySQL database. |
| -u user | Username with which to log into MySQL database. Default is"db_user". |
| -d database | Which database to push the values into. Default is "strawberry". |
| -t table | Which table in the database to push the values into. Default is "strawberry_fruit_v2_0". |

Table S3: Command-line options accepted by import_transcriptome.py. The script is designed to import transcriptomic data from specially-formated Excel documents so a MySQL database for use by eFP.
